# Clinicopathologic features and long-term prognosis of hepatitis B virus-associated glomerulonephritis: a retrospective cohort study

**DOI:** 10.1007/s40620-023-01685-x

**Published:** 2023-07-31

**Authors:** Hailing Lu, Yu Li, Maxiu Lai, Tianjun Guan, Yinghao Yu, Zhiyong Zheng, Yongze Zhuang

**Affiliations:** 1 Department of Nephrology, 900 Hospital of the Joint Logistics Team, PLA, Fuzhou General Clinical Medical College of Fujian Medical University, 156 Xierhuanbei Road, Fuzhou, 350025 Fujian China; 2 Department of Nephrology, Zhongshan Hospital Affiliated to Xiamen University, Xiamen, China; 3 Department of Pathology, 900 Hospital of the Joint Logistics Team, PLA, Fuzhou General Clinical Medical College of Fujian Medical University, Fuzhou, China

**Keywords:** Hepatitis B virus-associated glomerulonephritis, Membranous nephropathy, Prognosis, Predictor

## Abstract

**Background:**

Hepatitis B virus-associated glomerulonephritis is a common form of secondary glomerulonephritis in China. However, the clinicopathological features and long-term prognosis of Hepatitis B virus-associated Glomerulonephritis remain only partially known.

**Methods:**

Biopsy-proven Hepatitis B virus-associated Glomerulonephritis patients were enrolled between November 1994 and December 2013 at our center. The composite endpoints were doubling serum creatinine, end-stage renal disease, or death from renal disease during follow-up. The clinicopathological features and predictors of the long-term prognosis of Hepatitis B virus-associated Glomerulonephritis patients were explored.

**Results:**

The median age of the 259 Hepatitis B virus-associated Glomerulonephritis patients was 31.0 years (IQR 24.0–40.0), and 71.0% were males. Among the patients, 45.2% presented with nephrotic syndrome, and 45.9% presented with proteinuria combined with hematuria. The two most prevalent pathological patterns were IgA nephropathy (27.0%) and membranous nephropathy (27.0%). The mean follow-up period was 68.8 ± 46.9 months. The 3-, 5-, and 10-year clinical event-free survival rates were 93.4%, 85.2%, and 70.3%, respectively. Multivariable Cox regression analysis showed that hypertension (HR 2.580, 95% CI 1.351–4.927, *P* = 0.004), hyperuricemia (HR 2.101, 95% CI 1.116–3.954, *P* = 0.021), glomerulosclerosis (*P* = 0.001), and intrarenal arterial lesions (*P* = 0.041) were independent predictors of composite clinical event endpoint. Patients in the antiviral therapy group exhibited a significantly better prognosis compared to those who received no antiviral therapy (log-rank *χ*^2^ = 5.772, *P* = 0.016).

**Conclusion:**

Hepatitis B virus-associated Glomerulonephritis has specific clinicopathologic features and should not be considered a benign disease in adults. Hypertension, hyperuricemia, glomerulosclerosis, and intrarenal arterial lesions were independent predictors of the long-term prognosis in Hepatitis B virus-associated Glomerulonephritis patients. Antiviral therapy could be effective in improving the long-term prognosis of Hepatitis B virus-associated Glomerulonephritis patients.

**Graphic abstract:**

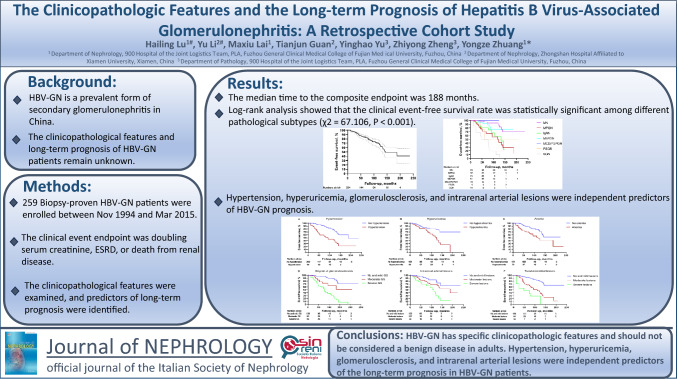

**Supplementary Information:**

The online version contains supplementary material available at 10.1007/s40620-023-01685-x.

## Introduction

Many studies have demonstrated that Hepatitis B virus-associated glomerulonephritis (HBV-GN) is the most prevalent extrahepatic manifestation of HBV infection. Hepatitis B virus-associated glomerulonephritis was first reported in 1971 by Combes et al. [1]. Since then, the relationship between HBV infection and nephropathy has received widespread attention. Recently, China has made great progress with universal vaccination for hepatitis B, which has significantly decreased the prevalence of HBV-GN. However, HBV-GN is still a common form of secondary glomerulonephritis among the Chinese population [2, 3].

Hepatitis B virus antigen deposition in the glomeruli suggests that complement activation induced by immune complex deposition plays a critical role in HBV-GN glomerular injury [4]. In most patients, HBV-GN presents as a chronic latent disease manifesting as nephrotic syndrome, while in others, it presents mild to moderate proteinuria and hematuria [5]. Many studies have reported that patients with HBV infection have different types of glomerulonephritis characterized by their pathological patterns, including membranous nephropathy (MN), membranoproliferative glomerulonephritis (MPGN), mesangial proliferative glomerulonephritis (MsPGN), IgA nephropathy (IgAN), minimal change disease (MCD), and focal segmental glomerulosclerosis (FSGS) [6–8].

Several studies have reported that spontaneous remission of HBV-associated membranous nephropathy (HBV-MN) is common in children, but rare in adults [6, 7, 9]. Although HBV-GN is common among the Asian population, including China, few studies focusing on the clinicopathological features and the long-term prognosis of HBV-GN in Asian populations have been conducted thus far. Therefore, this retrospective cohort study aims to elucidate the clinicopathological features, the long-term prognosis, and risk factors for the clinical event endpoint in 259 HBV-GN patients over ten years at our center.

## Methods

### Patients

This retrospective cohort study included 333 consecutive adult patients diagnosed with HBV-GN using renal biopsy at Fuzhou General Hospital of Nanjing Military Command (currently referred to as 900 Hospital of the Joint Logistics Team, PLA, Fuzhou General Clinical Medical College of Fujian Medical University). This study analyzed clinical data of patients who attended the center for treatment between November 1994 and December 2013. Patients with a follow-up period of more than 12 months were included in the research as of March 2015. Patients were excluded from this study if (1) they had incomplete clinical and pathologic data (*n* = 5), (2) were lost to follow-up (*n* = 57), and (3) the follow-up was less than 12 months (*n* = 12). The diagnostic criteria for HBV-GN were (1) HBV antigen-positive serum, (2) glomerular nephritis, excluding other secondary glomerular diseases, and (3) HBV antigens detected in kidney tissue by immunohistochemical staining [10]. On the basis of the inclusion and exclusion criteria, 74 patients were excluded, and 259 were included in our study.

### Clinical data collection

The demographic and laboratory characteristics of subjects include age, gender, systolic blood pressure, diastolic blood pressure, uric acid, hemoglobin, serum creatinine, estimated glomerular filtration rate (eGFR), and chronic kidney disease (CKD) stages. The study also recorded the patient’s serum albumin, 24-h urinary protein, alanine transaminase, aspartate transaminase, and HBV serum markers. In addition, we evaluated anemia following the World Health Organization (WHO) criteria [11], and eGFR was evaluated using the CKD-EPI formula [12]. After determining the laboratory characteristics of the patients, the patients were divided into four categories according to clinical manifestations: nephrotic syndrome, proteinuria alone, proteinuria combined with hematuria, and hematuria alone.

Medications including antiviral drugs, renin-angiotensin system (RAS) blockers, corticosteroids, and additional immunosuppressive agents were recorded. A total of 179 HBV-GN patients were treated with RAS blockers, except in the case of contraindications and no indication for RAS blocker use. One hundred sixty-one HBV-GN patients had received antiviral therapy, including 124 patients who had received antiviral therapy alone and 37 patients who had received combined antiviral and immunosuppressant therapy (corticosteroids and/or immunosuppressive agents). In addition, for patients who had received combined antiviral and immunosuppressant therapy, the antiviral therapy continued for 6 months after the withdrawal of immunosuppressant therapy.

### Histologic evaluation

**Fig. 1:**
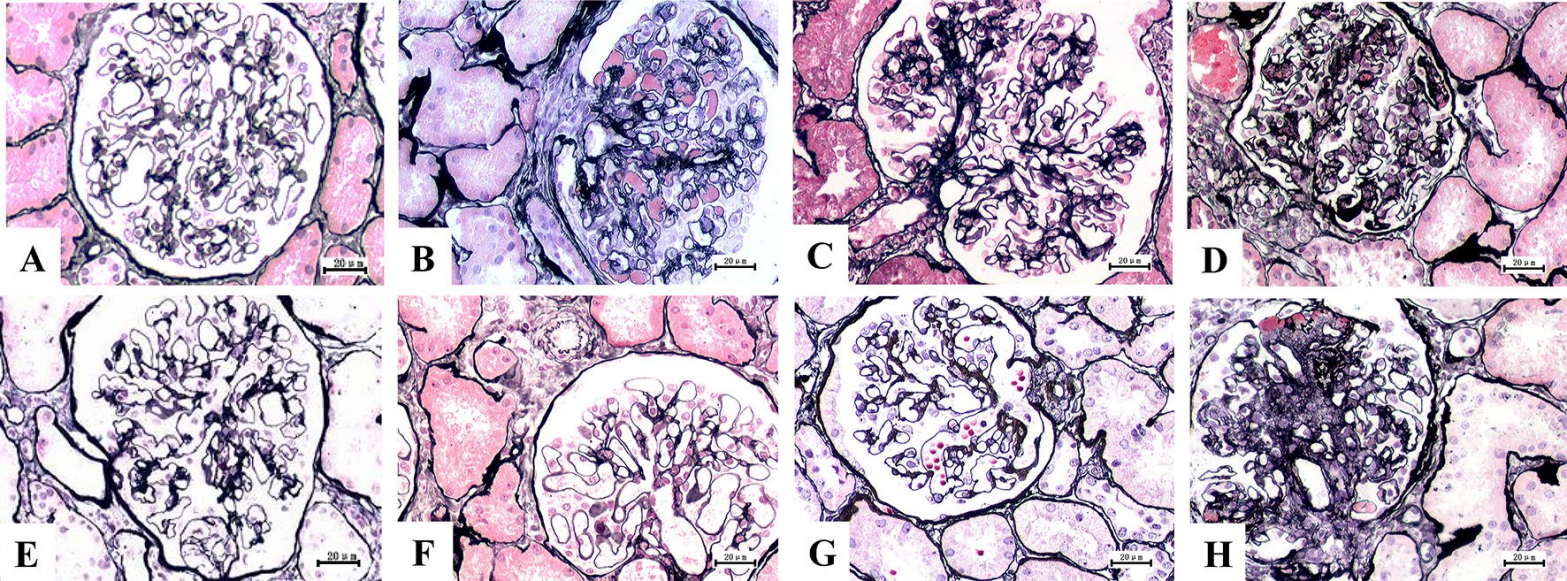
Pathologic subtypes of HBV-GN patients (× 400). Scale bar: 20 μm. **A** HBV-MN. **B** HBV-MPGN. **C** HBV-MsPGN. **D** HBV-IgAN. **E** HBV-FSGS. **F** HBV-FsPGN. **G** HBV-MCD. **H** HBV-SGN

The histological findings of the renal biopsies were classified following the WHO's revised Pathological Categories of Renal Glomerular Diseases [13]. This study examined eight pathological types: MN, MPGN, MsPGN, IgAN, MCD, FSGS, focal segmental proliferative glomerulonephritis (FsPGN), and sclerosing glomerulonephritis ([SGN]: 50–75% of glomeruli showed sclerosis, while the remnant glomeruli showed proliferation and segmental sclerosis), as reported in Fig. 1. The Katafuchi et al. [10, 14] criteria were used to score the presence of glomerulosclerosis, intrarenal arterial lesions, and tubulointerstitial lesions. The Katafuchi et al. criteria classify glomerulosclerosis into four levels: 0% = no glomerulosclerosis; < 25% = mild glomerulosclerosis; 25–50% = moderate glomerulosclerosis; and > 50% = severe glomerulosclerosis. Similarly, intrarenal arterial lesions, including intrarenal arterial-arteriolar wall thickening, arterial wall sclerosis, onion-skin change, and hyaline change, were scored as follows: 0 = no arterial lesions; 1–2 = mild arterial lesions; 3–4 = moderate arterial lesions; and 5–10 = severe arterial lesions. The tubulointerstitial lesions, including tubular atrophy, interstitial fibrosis, and inflammatory cell infiltration, were classified as follows: 0 = no tubulointerstitial lesions; 1–3 = mild tubulointerstitial lesions; 4–6 = moderate tubulointerstitial lesions; 7–9 = severe tubulointerstitial lesions. Biopsy specimens containing fewer than ten glomeruli were excluded from the analyses. This study used two pathologists, blinded to the patient’s clinical data, to examine and score the biopsy specimens.

### Endpoint event definition

The composite clinical event endpoint was defined as doubling serum creatinine, end-stage renal disease, or death from renal disease during follow-up. On the other hand, “Death from renal disease” was defined as death caused by a complication of renal failure (e.g., hyperkalemia, heart failure, arrhythmia, and cerebrovascular events), excluding death from non-renal causes (e.g., car accident, tumor).

### Statistical analyses

Categorical data were expressed as frequencies and percentages. The normally distributed continuous data were presented as the mean ± standard deviation, and non-normally distributed continuous data were presented as the median and interquartile range (IQR). Categorical data were analyzed using the chi-squared (*χ*^2^ test) or Fisher’s exact test as appropriate. Continuous data were compared using the *t*-test, Mann–Whitney *U*-test, Kruskal-Walls *H*-test, or one-way ANOVA as appropriate. This study used Spearman’s rank correlation coefficient to analyze correlations. In addition, the cumulative clinical event-free survival rate was estimated using the Kaplan–Meier method and compared using the log-rank test. Hazard ratios (HRs) were calculated using the Cox proportional hazards model with 95% confidence intervals (CIs) to identify the risk factors for long-term prognosis. In this study, factors with a *p*-value greater than 0.05 in the univariate analysis were included in the multivariate Cox regression model. This study combined patients with MCD and FsPGN because their sample size was small and had similar pathological features. Statistical analyses were performed using SPSS version 22.0 (Chicago, IL, USA). The significance level was set at *p* < 0.05 (2-tailed).

## Results

### Demographic and laboratory characteristics

The study included 184 males and 75 females, with a mean follow-up of 68.8 ± 46.9 months. The median age of the participants was 31.0 years. The baseline characteristics of the patients are presented in Table 1. The mean serum albumin level was 2.97 ± 0.90 g/dL. The median eGFR and urinary protein excretion was 99.4 mL/min/1.73 m^2^ (IQR 68.2–120.6) and 2.0 g/day (IQR 1.0–3.0), respectively. Hypertension, hyperuricemia, and anemia occurred in 57 (22.0%), 93 (35.9%), and 70 (27.0%) of the participants, respectively. Regarding HBV serum markers, patients were predominantly HBsAg + /HBeAg + /HBcAb + (57.5%) and HBsAg + /HBeAb + /HBcAb + (31.7%).

**Table 1:** Baseline characteristics of patients with HBV-GN

Characteristics	Total (*n* = 259)	Antiviral therapy group (*n* = 161)	No antiviral therapy group (*n* = 98)	*P* value
Demographic characteristics				
Age, years	31.0 (24.0–40.0)	32.0 (23.5–40.0)	31.0 (25.0–41.0)	0.822
Male, *n* (%)	184 (71.0)	112 (69.6)	72 (73.5)	0.502
Hypertension, *n* (%)	57 (22.0)	32 (19.9)	25 (25.5)	0.288
SBP, mmHg	126.9 ± 16.5	126.1 ± 16.1	128.1 ± 17.2	0.366
DBP, mmHg	80.4 ± 10.7	80.1 ± 11.6	81.0 ± 9.1	0.522
Follow-up period, months	68.8 ± 46.9	61.9 ± 43.4	80.2 ± 50.2	0.002
Laboratory characteristics				
Serum albumin, g/dL	2.97 ± 0.90	2.87 ± 0.83	3.13 ± 0.99	0.023
Serum creatinine, μmol/L	84.0 (64.0–106.0)	80.0 (62.5–102.5)	92.5 (70.5–122.3)	0.009
Blood urea nitrogen, mmol/L	5.4 (4.0–7.1)	5.4 (4.0–7.2)	5.3 (4.1–7.0)	0.969
eGFR, mL/min/1.73 m^2^	99.4 (68.2–120.6)	106.1 (75.4–120.8)	89.8 (64.3–118.3)	0.020
Hyperuricemia, *n* (%)	93 (35.9)	57 (35.4)	36 (36.7)	0.829
Uric acid, μmol/L	384.0 (317.0–452.0)	382.0 (318.5–448.5)	386.5 (311.5–456.0)	0.947
Anemia, *n* (%)	70 (27.0)	38 (23.6)	32 (32.7)	0.112
UP, g/day	2.0 (1.0–3.0)	2.81 (0.9–5.1)	2.0 (0.9–4.6)	0.354
ALT > 50 U/L, *n* (%)	66 (25.5)	46 (28.6)	20 (20.4)	0.144
AST > 40 U/L, *n* (%)	66 (25.5)	48 (29.8)	18 (18.4)	0.040
HBV serum markers, *n* (%)				< 0.001
HBsAg + , HBeAg + , HBcAb +	149 (57.5)	115 (71.4)	34 (34.7)	
HBsAg + , HBeAb + , HBcAb +	82 (31.7)	35 (21.7)	47 (48.0)	
HBsAg + , HBcAb +	18 (6.9)	8 (5.0)	10 (10.2)	
HBsAg +	10 (3.9)	3 (1.9)	7 (7.1)	
RAS blockers	179 (69.1)	112 (69.6)	67 (68.4)	0.840
Pathological types				0.043
MN	70 (27.0)	47 (29.2)	23 (23.5)	
MPGN	31 (12.0)	19 (11.8)	12 (12.2)	
IgAN	70 (27.0)	41 (25.5)	29 (29.6)	
MsPGN	51 (19.7)	36 (22.4)	15 (15.3)	
MCD/FsPGN	18 (6.9)	12 (7.5)	6 (6.1)	
FSGS	5 (1.9)	0 (0)	5 (1.9)	
SGN	14 (5.4)	6 (3.7)	8 (8.2)	

*SBP* systolic blood pressure, *DBP* diastolic blood pressure, *eGFR* estimated glomerular filtration, *UP* urinary protein, *ALT* alanine transaminase, *AST* aspartate transaminase

### Histological characteristics and correlations among variables

The most common pathological types were IgAN (27.0%) and MN (27.0%) in HBV-GN patients, as shown in Table 1. Eighty-five (32.8%) specimens were evaluated as no glomerulosclerosis, 73 (28.2%) as mild, 53 (20.5%) as moderate, and 48 (18.5%) as severe. Sixty-nine (26.6%) specimens were evaluated as no intrarenal arterial lesions, 34 (13.1%) as mild, 123 (47.5%) as moderate, and 33 (12.7%) as severe. In addition, arterial arteriolar wall thickening was present in 92.1% of the patients with intrarenal arterial lesions, and arterial wall sclerosis, hyaline change, and onion-skin change were seen in 63, 21, and 4 cases, respectively. Thirty-nine (15.1%) specimens were evaluated as no tubulointerstitial lesions, 154 (59.5%) as mild, 53 (20.5%) as moderate, and 13 (5.0%) as severe. A strong correlation was found between glomerulosclerosis and tubulointerstitial lesions (*r* = 0.614, *P* < 0.001), a moderate correlation was found between intrarenal arterial lesions and glomerulosclerosis (*r* = 0.371, *P* < 0.001), and also between intrarenal arterial lesions and tubulointerstitial lesions (*r* = 0.408, *P* < 0.001).

### Correlations between renal pathological types and clinical manifestations in HBV-GN patients

The most common pathological types were IgAN (27.0%) and MN (27.0%), followed by MsPGN, MPGN, MCN/FsPGN, SGN, and FSGS. Most patients presented with nephrotic syndrome (45.2%) or proteinuria combined with hematuria (45.9%), while only a small proportion presented with proteinuria alone (6.6%) or hematuria alone (2.3%). Patients with nephrotic syndrome had a higher MN, MPGN, MCD/FsPGN prevalence, and lower IgAN prevalence than proteinuria combined with the hematuria group, as shown in Table 2.

**Table 2:** The relationship between clinical categories and pathological types of HBV-GN

Pathological type	Clinical category				Total, *n* (%)
	NS	P	P + H	H	
MN	41	7	22^a^	0	70 (27.0)
MPGN	21	0	10^a^	0	31 (12.0)
IgAN	15	2	49^a^	4	70 (27.0)
MsPGN	22	1	26	2	51 (19.7)
MCD/FsPGN	12	2	4^a^	0	18 (6.9)
FSGS	2	0	3	0	5 (1.9)
SGN	4	5	5	0	14 (5.4)
Total, n (%)	117 (45.2)	17 (6.6)	119 (45.9)	6 (2.3)	259 (100.0)

*NS* nephrotic syndrome, *P* proteinuria alone, *H* hematuria alone, *P* + *H* proteinuria combined with hematuria

^a^*P* < 0.05 vs. NS group

### Different pathological types can predict HBV-GN prognosis

**Fig. 2:**
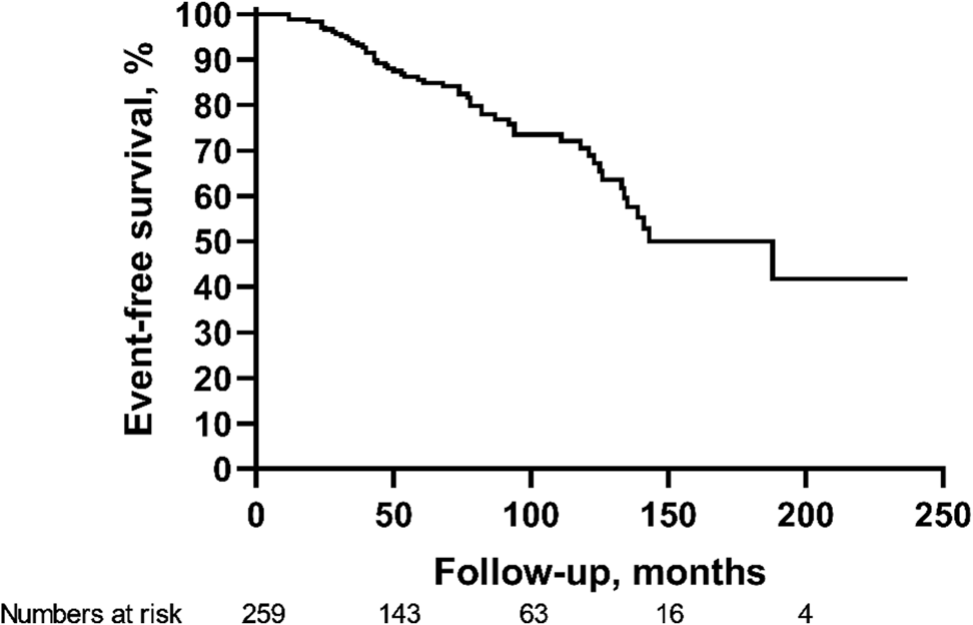
Long-term prognosis of HBV-GN patients. The median time to composite endpoint was 188 months

During the follow-up period, nine (3.5%) patients experienced a doubling of serum creatinine, 27 (10.4%) patients had end-stage renal disease, and 18 (6.9%) patients died from renal disease. In addition, the study also found that five patients died from non-renal causes, including liver cancer (*n* = 1), leukemia (*n* = 1), and car accidents (*n* = 3). The cumulative 3-, 5-, and 10-year clinical event-free survival rates were 93.4%, 85.2%, and 70.3%, respectively. The median time to the composite endpoint was 188 months, as shown in Fig. 2.

**Fig. 3:**
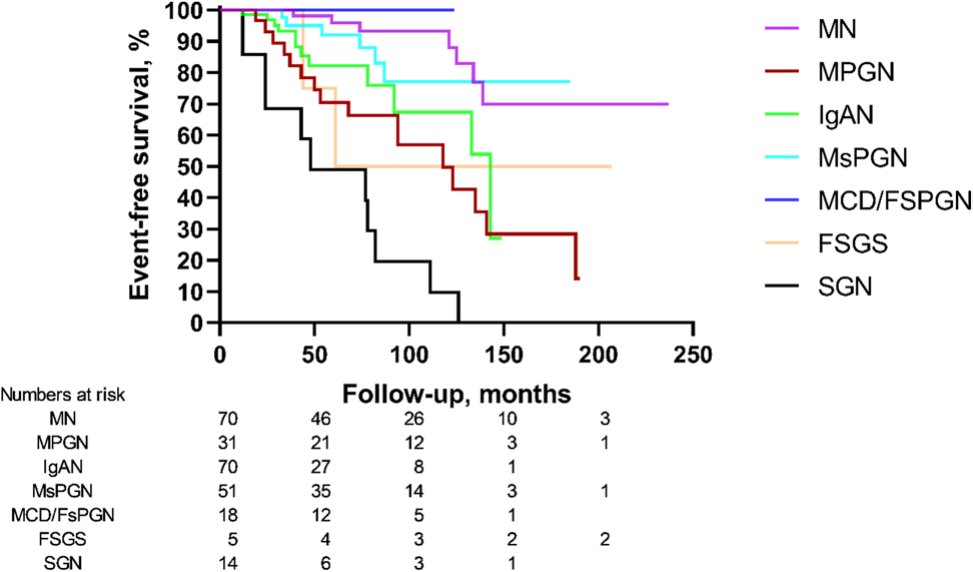
Long-term prognosis of patients according to different pathological subtypes. Log-rank analysis showed that the clinical event-free survival rate was statistically significant among different pathological subtypes (*χ*^2^ = 67.106, *P* < 0.001)

The composite clinical event endpoint was found in 0% of MCD/FsPGN, 10.0% of MN, 11.8% of MsPGN, 17.1% of IgAN, 40.0% of FSGS, 51.6% of MPGN, and 78.6% of SGN subtypes. In the Kaplan–Meier analysis, the clinical event-free survival rate was statistically significant among different pathological subtypes (log-rank *χ*^2^ = 67.106, *P* < 0.001), as shown in Fig. 3.

The composite clinical event endpoint was found in 23.1% of patients in the nephrotic syndrome group, 23.5% in the proteinuria alone group, 19.3% in the proteinuria combined with hematuria group, and in 0% of the hematuria alone group. In the Kaplan–Meier analysis, the clinical event-free survival rate was not statistically significant among the four clinical categories (log-rank *χ*^2^ = 1.777, *P* = 0.620).

### Hypertension, hyperuricemia, glomerulosclerosis, and intrarenal arterial lesions were independent predictors of HBV-GN prognosis

**Fig. 4:**
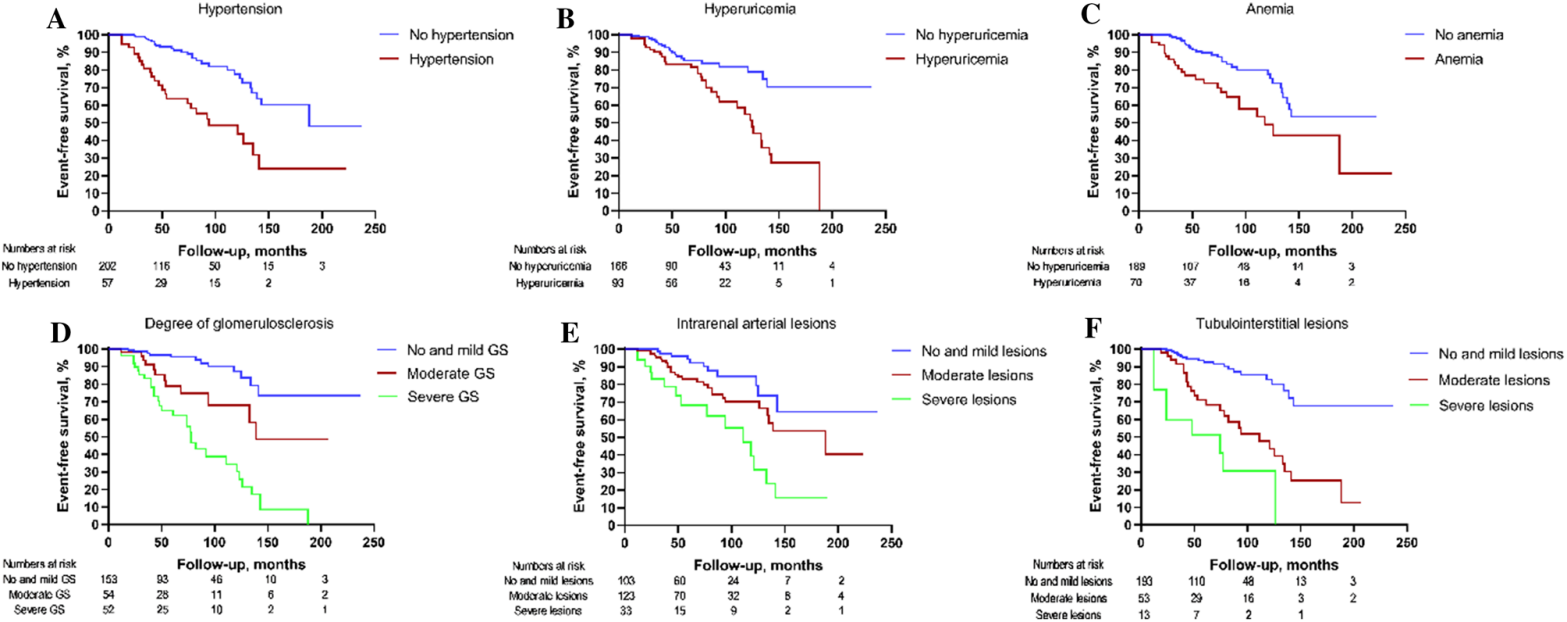
The cumulative event-free survival rate in HBV-GN patients categorized by hypertension, hyperuricemia, anemia, glomerulosclerosis, intrarenal arterial lesions, and tubulointerstitial lesions. Log-rank analysis showed that there were significant differences in event-free survival rate between **A** hypertension and no hypertension (*χ*^2^ = 24.600, *P* < 0.001), **B** hyperuricemia and no hyperuricemia (*χ*^2^ = 13.159, *P* < 0.001), **C** anemia and no anemia (*χ*^2^ = 12.468, *P* < 0.001) and among (**D**) no and mild degree of glomerulosclerosis (GS), moderate degree of GS and severe degree of GS (*χ*^2^ = 57.240, *P* < 0.001), **E** no and mild degree of intrarenal arterial lesions, moderate degree of intrarenal arterial lesions and severe degree of intrarenal arterial lesions (*χ*^2^ = 18.435, *P* < 0.001), **F** no and mild degree of tubulointerstitial lesions, moderate degree of tubulointerstitial lesions and severe degree of tubulointerstitial lesions (*χ*^2^ = 54.449, *P* < 0.001)

Kaplan–Meier analysis revealed that hypertension, hyperuricemia, anemia, glomerulosclerosis, intrarenal arterial lesions, and tubulointerstitial lesions were associated with a lower cumulative clinical event-free survival rate, as shown in Fig. 4.

Univariate Cox regression analysis revealed that hypertension, hyperuricemia, anemia, increased serum creatinine (per 10 μmol/L change), glomerulosclerosis, intrarenal arterial lesions, and tubulointerstitial lesions were significantly associated with clinical event-free survival. On the other hand, multivariate Cox regression analysis revealed that hypertension (HR 2.580, 95% CI 1.351–4.927, *P* = 0.004), hyperuricemia (HR 2.101, 95% CI 1.116–3.954, *P* = 0.021), glomerulosclerosis (*P* = 0.001), and intrarenal arterial lesions (*P* = 0.041) were independent risk factors. In addition, the risk of developing clinical events was associated with the severity of glomerulosclerosis and intrarenal arterial lesions, as shown in Table 3.

**Table 3:** Univariate and multivariate analysis of risk factors for developing clinical events

	Univariate analysis			Multivariate analysis		
	HR	95% CI	*P* value	HR	95% CI	*P* value
Age	1.011	0.987–1.035	0.377			
Gender						
Male	1.000					
Female	0.502	0.236–1.066	0.073			
Hypertension						
Negative	1.000					
Positive	3.561	2.082–6.091	< 0.001	2.580	1.351–4.927	0.004
Anemia			< 0.001			0.160
No	1.000			1.000		
Mild	2.123	1.177–3.830	0.012	1.907	0.980–3.712	0.057
Moderate and severe	5.128	2.229–11.798	< 0.001	1.287	0.482–3.438	0.615
Hyperuricemia						
Negative	1.000					
Positive	2.656	1.533–4.602	< 0.001	2.101	1.116–3.954	0.021
Serum creatinine (per 10 μmol/l change) (every 10 umol/L)	1.018	1.010–1.026	< 0.001	0.995	0.982–1.007	0.400
Glomerulosclerosis			< 0.001			0.001
No and mild	1.000			1.000		
Moderate	3.280	1.470–7.317	0.004	3.376	1.438–7.927	0.005
Severe	8.826	4.512–17.267	< 0.001	5.370	2.182–13.215	< 0.001
Intrarenal arterial lesions			< 0.001			0.041
No and mild	1.000			1.000		
Moderate	2.008	0.999–4.037	0.050	1.214	0.566–2.604	0.619
Severe	4.816	2.209–10.503	< 0.001	2.623	1.101–6.247	0.029
Tubulointerstitial lesions			< 0.001			0.881
No and mild	1.000			1.000		
Moderate	4.131	2.291–7.448	< 0.001	1.004	0.436–2.313	0.993
Severe	10.651	4.788–23.694	< 0.001	1.344	0.342–5.275	0.672

### Antiviral therapy may help to improve the prognosis of HBV-GN patients

**Fig. 5:**
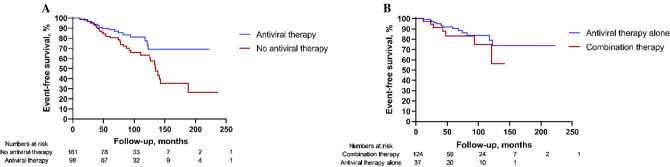
Long-term prognosis of HBV-GN patients categorized by **A** the antiviral therapy group and no antiviral therapy group, and **B** the antiviral therapy alone group and combined antiviral and immunosuppressant therapy group. Log-rank analysis showed that there was a significant difference between the antiviral therapy group and no antiviral therapy group (*χ*^2^ = 5.772, *P* = 0.016), but no significant difference between the antiviral therapy alone group and combined antiviral and immunosuppressant therapy group (*χ*^2^ = 1.169, *P* = 0.280)

The demographic and clinical data at baseline of both the antiviral therapy group and no antiviral therapy group are shown in Table 1. There was no significant difference in RAS blocker use. Compared with those in the no antiviral therapy group, the patients in the antiviral therapy group had shorter follow-up periods and fewer FSGS patients. Patients in the antiviral therapy group exhibited a significantly better prognosis compared to those in the no antiviral therapy group (log-rank *χ*^2^ = 5.772, *P* = 0.016), as shown in Fig. 5A.

Compared with those in the combined antiviral and immunosuppressant therapy group, the antiviral therapy alone group had more FSGS patients. Other detailed baseline characteristics are presented in Supplementary Table 1. There was no significant difference in the long-term prognosis between the antiviral therapy alone group and the combined antiviral and immunosuppressant therapy group (log-rank *χ*^2^ = 1.169, *P* = 0.280), as shown in Fig. 5B.

## Discussion

In this study, we explored the risk factors influencing the development of clinical events in 259 HBV-GN patients in China. It represents the largest number of HBV-GN cases with follow-up information in the world. This study found that the 3-, 5-, and 10-year clinical event-free survival rates were 93.4%, 85.2%, and 70.3%, respectively. The median time for the patients to reach the composite clinical event endpoint was 188 months. To our knowledge, only one study has explored the prognosis of HBV-MN in adult patients [6]. With an average follow-up of 73 months, they found that 6 patients (28.6%) with HBV-MN developed renal failure, and 2 (9.5%) required maintenance dialysis.

Our study identified IgAN (27.0%) and MN (27.0%) as the most common pathological types of HBV-GN. These findings were consistent with the results of studies conducted in Thailand, Hong Kong, and South Africa [6–8]. On the contrary, one study in Japan reported that MPGN is the prevalent pathological type of HBV-GN [15]. In addition, another study in China reported that MsPGN is the prevalent pathological type of HBV-GN [16].

Many studies have demonstrated that the prognosis of HBV-GN depends on the pathological subtype. Our study found that patients with MCD/FsPGN had an excellent prognosis, and patients with MN and MsPGN had a better prognosis than those with IgAN. However, patients with MPGN, FSGS, and SGN had poor long-term prognoses, possibly due to a higher incidence of renal insufficiency and hypertension. Zhang et al. [16] reported that patients with CKD stage 3 and above accounted for 11.6% (38/329) of HBV-GN patients in northeastern Chinese adults. However, patients with MPGN (12/38, 31.6%) and MsPGN (8/38, 21.1%) were more prone to develop renal failure among the 38 cases. Different regions, inclusion criteria, immunosuppressive and antiviral therapy indications, and RAS blockers or endpoint definitions may account for these discrepancies.

In this study, the nephrotic syndrome group and proteinuria combined with hematuria group accounted for the largest number of HBV-GN patients (236/259, 91.1%). Patients with IgAN often presented with persistent hematuria and proteinuria, whereas most HBV-GN patients with MN exhibited nephrotic syndrome with or without renal dysfunction, which was consistent with previous studies [6, 8]. Usually, patients with nephrotic syndrome have a worse kidney prognosis than those without nephrotic syndrome. However, this study found that the long-term prognosis of patients with different clinical categories was similar, suggesting that the HBV-GN prognosis was based more on pathological results than on clinical presentation.

Univariate Cox regression analysis demonstrated that hypertension, hyperuricemia, anemia, increased serum creatinine, glomerulosclerosis, intrarenal arterial lesions, and tubulointerstitial lesions were independent risk factors for composite clinical events endpoint. These results were concordant with the Kaplan–Meier survival analysis. After adjusting various clinical and pathological factors in the multivariate Cox model, hypertension, hyperuricemia, glomerulosclerosis, and intrarenal arterial lesions remained independent predictors, whereas serum creatinine, anemia status, and tubulointerstitial lesions did not. This result is justifiable, considering that serum creatinine levels are affected by many factors, such as diuretic use, muscle mass, and residual kidney function. There were many reasons for excluding tubulointerstitial lesions from the multivariable analysis. First, previous studies demonstrated that tubulointerstitial lesions were an independent adverse prognostic factor for glomerular disease patients [17, 18]; however, intrarenal arterial lesions were not included in the analysis. Second, from the perspective of pathophysiology, renal tubules and interstitial tissues are supplied by the vasa recta branching from efferent arterioles. Thus, the intrarenal arterial lesions and glomerulosclerosis lead to ischemic and hypoxic injury and promote progression of tubulointerstitial lesions. Third, a previous study confirmed that intrarenal arterial lesions and glomerulosclerosis are associated with an increased risk of tubulointerstitial lesions [19]. In conclusion, these results indicate that histopathological indicators of intrarenal arterial lesions and glomerulosclerosis are associated with poor prognosis in HBV-GN patients.

Previous studies have found that the prevalence and severity of CKD are associated with uric acid levels [20, 21]. Our previous study demonstrated that hyperuricemia was associated with hypertension, elevated serum creatinine, glomerulosclerosis, and renal tubular interstitial injury in HBV-GN [5]. This cohort study identified glomerulosclerosis as an unfavorable prognostic risk factor. Patients with concomitant glomerulosclerosis in pathological findings, such as FSGS and SGN, had a poor prognosis. Our previous study showed that intrarenal arterial lesions were correlated with higher blood pressure, reduced renal function, and more severe interstitial injury in HBV-GN patients [10]. The study also found that intrarenal arterial lesions strongly impacted the renal prognosis of HBV-GN [10]. These findings were reported for other renal diseases, such as IgAN and lupus nephritis [22, 23].

To date, there are no reliable data on the long-term prognostic effect of the treatment protocol in patients with HBV-GN. A previous meta-analysis showed that antiviral therapy against HBV infection significantly reduced proteinuria in HBV-MN patients [24]. Our study confirmed that antiviral therapy significantly improved the long-term prognosis of HBV-GN patients, but the baseline differences may have induced a bias. In addition, a meta-analysis found that combined antiviral and immunosuppressant therapy could reduce proteinuria in HBV-GN patients without HBV reactivation [25]. In our study, patients in the combined antiviral and immunosuppressant therapy subgroup had less pathological severity compared with the antiviral therapy alone group, however, there was no additional benefit observed in long-term prognosis in patients treated with combined antiviral and immunosuppressant therapy compared with antiviral therapy alone.

This study has several limitations. First, it combined several variables or groups with small sample sizes or similar pathological features, thus reducing the statistical power. Second, this is a single-center retrospective study.

In conclusion, this study explored the clinicopathological features and predictors of long-term prognosis in HBV-GN patients. Results showed that 29.7% of patients developed the composite clinical event endpoint within ten years. Hypertension, hyperuricemia, glomerulosclerosis, and intrarenal arterial lesions were independent predictors of the long-term prognosis in adult HBV-GN patients. Antiviral therapy could be effective in improving the long-term prognosis of HBV-GN patients.

### Supplementary Information

Below is the link to the electronic supplementary material.

Supplementary file1 (DOCX 26 KB)

## Data Availability

All data generated or analyzed in this study are included in this published article.
