# Supplementary material for: Clinicopathologic features and long-term prognosis of hepatitis B virus-associated glomerulonephritis: a retrospective cohort study
Source: J Nephrol. 2023 Jul 31;36(8):2335–44. doi: 10.1007/s40620-023-01685-x (PMC10638113; doi:10.1007/s40620-023-01685-x)
Supplement: Supplementary file 1 — Supplementary file1 (DOCX 26 KB) [file 40620_2023_1685_MOESM1_ESM.docx]

**Supplementary Table 1. Baseline characteristics of HBV-GN patients with antiviral therapy.**

| Characteristics | Total (n=161) | Combined antiviral and immunosuppressant therapy group (n=124) | Antiviral therapy alone group (n=37) | P value |
| --- | --- | --- | --- | --- |
| Demographic characteristics |  |  |  |  |
| Age, years | 32.0 (23.5–40.0) | 31.0 (23.0-40.0) | 33.0 (26.0-41.0) | 0.268 |
| Male, n (%) | 112 (69.6) | 87 (70.2) | 25 (67.6) | 0.763 |
| Hypertension, n (%) | 32 (19.9) | 23 (18.5) | 9 (24.3) | 0.440 |
| SBP, mmHg | 126.1 ± 16.1 | 125.5 ± 15.2 | 128.3 ± 18.9 | 0.356 |
| DBP, mmHg | 80.1 ± 11.6 | 79.9 ± 11.6 | 80.9 ± 11.7 | 0.646 |
| Follow-up period, months | 61.9 ± 43.4 | 61.3 ± 45.2 | 63.9 ± 37.5 | 0.752 |
| Laboratory characteristics |  |  |  |  |
| Serum albumin, g/dL | 2.97 ± 0.90 | 2.71 ± 0.79 | 3.41 ± 0.71 | <0.001 |
| Serum creatinine, μmol/L | 80.0 (62.5–102.5) | 80.5 (64.0-97.8) | 73.0 (59.5-106.5) | 0.771 |
| Blood urea nitrogen, mmol/L | 5.4 (4.0–7.2) | 5.6 (4.1-7.7) | 4.7 (3.8-6.2) | 0.071 |
| eGFR, mL/min/1.73 m^2^ | 106.1 (75.4–120.8) | 106.4 (76.9-120.0) | 102.4 (72.3-125.3) | 0.955 |
| Hyperuricemia, n (%) | 57 (35.4) | 49 (39.5) | 8 (21.6) | 0.046 |
| Uric acid, μmol/L | 382.0 (318.5–448.5) | 394.0 (324.0-461.8) | 343.0 (280.0-410.5) | 0.017 |
| Anemia, n (%) | 38 (23.6) | 28 (22.6) | 10 (27.0) | 0.576 |
| UP, g/day | 2.8 (0.9–5.1) | 3.7 (1.5-5.5) | 1.0 (0.4-1.9) | <0.001 |
| ALT > 50 U/L, n (%) | 46 (28.6) | 35 (28.2) | 11 (29.7) | 0.859 |
| AST > 40 U/L, n (%) | 48 (29.8) | 33 (26.6) | 15 (40.5) | 0.104 |
| HBV serum markers, n (%) |  |  |  | 0.432 |
| HBsAg+, HBeAg+, HBcAb+ | 115 (71.4) | 87 (70.2) | 28 (75.7) |  |
| HBsAg+, HBeAb+, HBcAb+ | 35 (21.7) | 30 (24.2) | 5 (13.5) |  |
| HBsAg+, HBcAb+ | 8 (5.0) | 5 (4.0) | 3 (8.1) |  |
| HBsAg+ | 3 (1.9) | 2 (1.6) | 1 (2.7) |  |
| RAS blockers | 112 (69.6) | 87 (70.2) | 25 (67.6) | 0.763 |
| Pathological types |  |  |  | 0.020 |
| MN | 47 (29.2) | 40 (32.3) | 7 (18.9) |  |
| MPGN | 19 (11.8) | 13 (10.5) | 6 (16.2) |  |
| IgAN | 41 (25.5) | 32 (25.8) | 9 (24.3) |  |
| MsPGN | 36 (22.4) | 29 (23.4) | 7 (18.9) |  |
| MCD/FsPGN | 12 (7.5) | 9 (7.3) | 3 (8.1) |  |
| SGN | 6 (3.7) | 1 (0.8) | 5 (13.5) |  |
| Clinical categories |  |  |  | <0.001 |
| Nephrotic syndrome | 82 (50.9) | 75 (60.5) | 7 (18.9) |  |
| Proteinuria alone | 11 (6.8) | 5 (4.0) | 6 (16.2) |  |
| Hematuria alone | 5 (3.1) | 1 (0.8) | 4 (10.8) |  |
| Proteinuria combined with hematuria | 63 (39.1) | 43 (34.7) | 20 (54.1) |  |
